# Cervical Cerclage to Prevent Intrauterine Balloon Prolapse

**DOI:** 10.1055/s-0042-1749427

**Published:** 2023-02-03

**Authors:** Toshifumi Suzuki, Jun Takeda, Rie Seyama, Shintaro Makino, Satoru Takeda, Atsuo Itakura

**Affiliations:** 1 Department of Obstetrics and Gynecology, Juntendo University Faculty of Medicine, Tokyo, Japan; 2 Department of Obstetrics and Gynecology, Keiai Hospital, Saitama, Japan; 3 Department of Obstetrics and Gynecology, Juntendo University Urayasu Hospital, Urayasu City, Chiba, Japan; 4 Aiiku Research Institute for Maternal, Child Health and Welfare, Tokyo, Japan

**Keywords:** cervical cerclage, intrauterine balloon tamponade, placenta previa, cervical dilation, intrauterine balloon prolapse

## Abstract

Intrauterine balloon prolapse sometimes occurs, and the intrauterine balloon must be reinserted. Furthermore, intrauterine balloon tamponade (IBT) failure can necessitate additional invasive procedures. We report a case of cervical cerclage with IBT for placenta previa with a cervical dilation. In our case, emergency cesarean section was performed at 35 + 4 weeks of gestation because of persistent hemorrhage. During the operation, we performed IBT to prevent further postpartum hemorrhage. However, immediately after the operation, uterine cervical dilatation was 6 cm, which resulted in cervical dilation and prolapse of the intrauterine balloon. Therefore, we performed cervical cerclage using absorbable sutures with IBT and blood transfusion. We speculated that the intrauterine balloon might have induced cervical canal ripening during the operation. Our case suggested that cervical cerclage with IBT is a useful method to prevent intrauterine balloon prolapse in cases with cervical dilation.

Intrauterine balloon tamponade (IBT) is an effective method to control intraoperative and postpartum hemorrhage. The mechanisms underlying achieving hemostasis are considered direct pressure to the bleeding point/area, and stimulating uterine contractions. 1 Of these mechanism, direct pressure to the bleeding area is important in cases of placenta previa because even appropriate uterine contractions do not always lead to hemostasis. It has been previously reported that the major cause of IBT failure is balloon prolapse. 2 In addition, uterine cervical dilation with placenta previa may initiate persistent massive bleeding. 2 3 Consequently, in some cases, the amount of bleeding increased, which led to disseminated intravascular coagulation. These cases could require additional invasive procedures, such as uterine artery embolization and emergency hysterectomy. However, invasive procedures have the potential to decrease fertility 4 ; therefore, measures to prevent balloon prolapse are important. 5 6 In 2011, Jain described the concept of the method of cerclage with IBT. 7 Here, we secondary describe a case of cervical cerclage with IBT for placenta previa that involved cervical dilation and we discuss the effect of IBT with cervical cerclage.

## Case Report

**Fig. 1:**
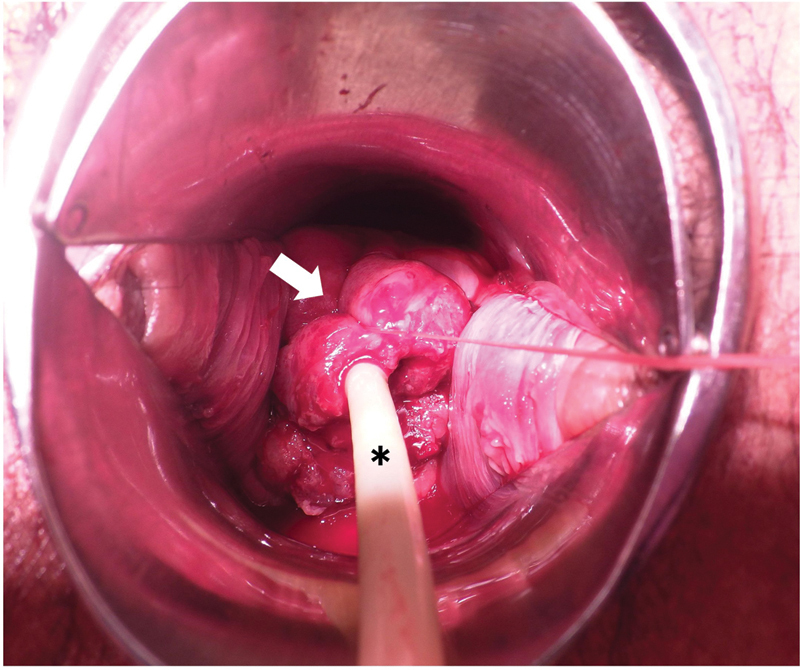
Cervical cerclage using absorbable surgical suture with a Fuji-Metro intrauterine balloon. The arrow indicates the cervical cerclage with absorbable suture. The asterisk indicates the Fuji-Metro intrauterine balloon.

The patient was a gravida 1, para 0, 36-year-old Japanese woman diagnosed with total placenta previa, who required maternal transport to our institute at 34 + 3 weeks of gestation. Emergency cesarean section was performed at 35 + 4 weeks of gestation because of persistent hemorrhage. Intraoperatively, to prevent postpartum hemorrhage, IBT was performed with a Fuji-Metro (Fuji Latex Co., Ltd., Tokyo, Japan) intrauterine balloon, originally made for cervical ripening. This device has no drainage port, and the inflation volume was 150 mL. The cesarean section was completed without blood transfusion, and with a blood loss volume of 1,704 mL, including amniotic fluid. Immediately after the operation, persistent massive hemorrhage occurred, with a low fibrinogen level of 196 mg/dL, uterine cervical dilatation of 6 cm, and balloon prolapse toward vagina was occurred, although uterine atony was not observed. Therefore, we performed cervical cerclage, similarly to the McDonald method, with IBT and with 300-mL inflation volume ( Fig. 1 ). In addition, the patient received 6 units each of red blood cells and fresh-frozen plasma, and cryoprecipitate derived from 8 units of fresh-frozen plasma before leaving the operating room. After cervical cerclage and with the balloon remaining in the uterus, the massive hemorrhage stopped. The final total blood loss was 2,554 mL. On postoperative day 1, the Fuji-Metro and cerclage sutures were removed. The patient had an uneventful postoperative course, and was discharged from hospital 5 days after the cesarean section.

## Discussion

In our case, cervical cerclage prevented balloon prolapse and controlled further hemorrhage. The success of IBT results from two factors: (1) direct compression of the bleeding point, and (2) preventing prolapse of the intrauterine balloon. 5 6 Jain first mentioned the concept of the method of cerclage with IBT. 7 In addition, recently, some techniques, such as the “fishing method” and “holding the cervix” in IBT with cervical cerclage have been reported for intraoperative and postpartum hemorrhage. These methods are completed using cervical laceration forceps or surgical sutures. 8 9 10 Holding the cervix using cervical laceration forceps is relatively cost-effective and involves a short operation time. However, the technique has disadvantages, such as local pain and prolapse of the cervical laceration forceps. In addition, in cases of recurrent rupture of uterine artery pseudoaneurysms, IBT might be necessary for more than 24 hours. 1 Therefore, using cervical laceration forceps could prevent early ambulation. In contrast, cervical cerclage can tighten the cervical canal surrounding the intrauterine balloon to prevent balloon prolapse. However, our method might promote bleeding from the uterine cervix, especially in case of placenta previa. Therefore, in the case of deep narrow surgical field by massive bleeding, other technique might be needed. To the best of our knowledge, there are few reports of cervical cerclage using sutures with IBT, although its concept was mentioned. 7 8 The novelty of our case report is that we used a Fuji-Metro balloon, which has no drain. Balloons without drainage ports permit tightening the cervical cerclage to prevent balloon prolapse more than when using balloons with drainage ports. The cerclage cannot be tightened as much with balloons with a drain because the port may become compressed. Therefore, our method differs from previous reports using Bakri balloons regarding the tightness of the cerclage. However, our method might delay in the recognition of intra-abdominal hemorrhage. In our case, we performed cervical cerclage with sutures without unbearable local pain or prolapse of the Fuji-Metro, although additional operation time was required owing to continuous bleeding ( Fig. 1 ). Further blood loss could be controlled by IBT with cervical cerclage and blood transfusion.

In our case, immediately after the cesarean section, uterine cervical dilatation occurred, although the cervical canal did not open during the operation. We speculated that the intrauterine balloon might have induced cervical canal ripening during the operation. Our case suggests that using a balloon without a drainage port with cervical cerclage is a useful method in cases of cervical dilation, especially for placenta previa because the balloon must be placed at the lower part of the uterine isthmus, which may lead to uterine cervical maturation.

In conclusion, the present case supports the concept that cervical cerclage with IBT is a useful method to prevent intrauterine balloon prolapse with cervical dilation. It is necessary to select some techniques according to the various situations.
